# Molecular Classification of Pesticides Including Persistent Organic Pollutants, Phenylurea and Sulphonylurea Herbicides

**DOI:** 10.3390/molecules19067388

**Published:** 2014-06-05

**Authors:** Francisco Torrens, Gloria Castellano

**Affiliations:** 1 Institut Universitari de Ciència Molecular, Universitat de València, Edifici d’Instituts de Paterna, P.O. Box 22085, E-46071 València, Spain; 2 Facultad de Veterinaria y Ciencias Experimentales, Universidad Católica de Valencia San Vicente Mártir, Guillem de Castro-94, E-46001 València, Spain; E-Mail: gloria.castellano@ucv.es

**Keywords:** periodic law, periodic property, periodic table, molecular classification

## Abstract

Pesticide residues in wine were analyzed by liquid chromatography–tandem mass spectrometry. Retentions are modelled by structure–property relationships. Bioplastic evolution is an evolutionary perspective conjugating effect of acquired characters and evolutionary indeterminacy–morphological determination–natural selection principles; its application to design co-ordination index barely improves correlations. Fractal dimensions and partition coefficient differentiate pesticides. Classification algorithms are based on *information entropy* and its production. Pesticides allow a structural classification by nonplanarity, and number of O, S, N and Cl atoms and cycles; different behaviours depend on number of cycles. The novelty of the approach is that the structural parameters are related to retentions. Classification algorithms are based on information entropy. When applying procedures to moderate-sized sets, excessive results appear compatible with data suffering a combinatorial explosion. However, *equipartition conjecture* selects criterion resulting from classification between hierarchical trees. Information entropy permits classifying compounds agreeing with principal component analyses. Periodic classification shows that pesticides in the same group present similar properties; those also in equal period, maximum resemblance. The advantage of the classification is to predict the retentions for molecules not included in the categorization. Classification extends to phenyl/sulphonylureas and the application will be to predict their retentions.

## 1. Introduction

Twenty-six billion litres of wine were produced worldwide and 24 billion litres, consumed in 2010 according to the International Organization of Vine and Wine. Wine, especially red wine, is rich in polyphenols (e.g., resveratrol, catechin, epicatechin), which are antioxidants that protect cells from oxidative damage caused by free radicals. Red-wine antioxidants inhibit cancer development, e.g., that of prostate cancer. Red-wine consumption presents heart-health benefits. Application of pesticides (e.g., fungicides, insecticides) to improve grape yields is common. However, pesticides permeate via the plant tissues and remain in harvested grapes/processed products (e.g., grape juice, wine). Because pesticides are a source of toxicants that are harmful to human beings it is important to test for their levels in grapes, juice and wine. Although EU has set maximum residue levels (MRLs) for pesticides in wine grapes of 0.01–10 mg·kg^−1^ it has not done so for wine. An EU-wine study revealed that 34 out of 40 bottles contained at least one pesticide. Average number was >4 pesticides per bottle while the highest number was 10. Pesticide analysis in red wine is challenging because of the complexity of the matrix that contains alcohol, organic acids, sugars, phenols and pigments, e.g., anthocyanins. Traditional red-wine sample preparation methods include liquid–liquid extraction (LLE) with organic solvents [1,2] and solid-phase extraction (SPE) with reversed-phase C18/polymeric sorbents [3,4,5]. However, LLE is labour-intensive, consumes large amounts of organic solvents and forms emulsions making difficult to separate organic/aqueous phases. In contrast, SPE demands more development. Solid-phase microextraction (SPME) [6,7], hollow-fibre liquid-phase microextraction [8] and stir-bar sorptive extraction (SBSE) [9] are lesser reproducible. Typical detections incorporate gas chromatography (GC), GC coupled to mass spectrometry (MS) (GC–MS) and liquid chromatography coupled to tandem MS (LC–MS–MS).

Quick, easy, cheap, effective, rugged and safe (QuEChERS) is a sample preparation method that was reported for pesticide-residue determination in vegetables/fruits [10]; it was used for pesticide/compound analysis in various food, oil and beverage matrices [11,12,13]; QuEChERS involves pesticide extraction from a sample with high water content into acetonitrile, with addition of salts to separate phases and partition the pesticides into the organic layer, which is followed by dispersive SPE (dSPE) to clean up various matrix co-extractives and achieve mixing of an aliquot of sample extract with sorbents prepacked in a centrifuge tube. Pesticide determination in red wine was reported [14]. Eight pesticides belonging to the insecticide (methamidophos, diazinone, pyrazophos, chlorpyrifos), fungicide (carbendazim, thiabendazole, pyrimethanil, cyprodinil, pyrazophos) and parasiticide (thiabendazole) classes were selected. Their polarities are different. Some are planar (carbendazim, thiabendazole, pyrimethanil, cyprodinil). Cyprodinil was most usually detected on grapes with chlorpyrifos, diazinone and methamidophos, frequent. Carbendazim was detected in three out of six red-wine samples. Occurrence and removal efficiency of pesticides in sewage treatment plants from Spanish, Mediterranean, Brazilian and other rivers were reviewed [15,16] and reported [17,18]. Transport of organic persistent microcontaminants associated with suspended particulate material in the Ebro River Basin was described [19,20]. Several researchers have reported the quantitative structure–activity/property relationships (QSAR/QSPR) of pesticides. The Benfenati group modelled the QSPR of the octanol/water partition coefficient of organometallic substances by optimal SMILES-based descriptors [21], QSAR of the toxicity of organic substances to *Daphnia magna* via freeware CORAL [22], and optimal descriptor as a translator of eclectic data into endpoint prediction and mutagenicity of fullerene as a mathematical function of conditions [23]. The Roy group modelled predictive chemometrics and three-dimensional toxicophore mapping of diverse organic chemicals causing bioluminescent repression of the bacterium genus *Pseudomonas* [24] and QSAR for toxicity of ionic liquids to *D. magna* analyzing aromaticity *vs.* lipophilicity [25].

The chromatographic retention time was correlated to the stationary and mobile phases of the system. In earlier publications the free energy of solvation and partition coefficients in methanol–water binary mixtures were analized [26]. Stationary phase was modelled in size-exclusion chromatography with binary eluents as a strategy in size-exclusion chromatography [27]. Stationary–mobile phase distribution coefficient for polystyrene standards was represented [28]. A new chemical index inspired by plastic evolution was presented [29] and applied to valence-isoelectronic series of aromatics [30]. QSPR of retention times of phenylureas [31,32] and pesticides [33] was described by plastic evolution. A simple computerized algorithm was proposed to be useful for establishing relationships between chemical structures and biosignificance [34,35]. Starting point is to use information entropy for pattern recognition. Entropy is formulated on basis of *similarity matrix* between two biochemical species. As entropy is weakly discriminating for classification, the more powerful concepts of *entropy production* and *equipartition conjecture* were introduced [36]. The aim of the present report is to find properties that distinguish pesticide structures according to retention times. The study applies a chemical index to pesticides. The goal is index usefulness validation via the capability to distinguish between pesticides, and interest as a predictive index for retention as compared with fractal dimensions and partition coefficients. Section 2 illustrates and discusses the results. Section 3 presents the computational method, including classification algorithm, information entropy, equipartition conjecture of entropy production and learning procedure. Finally, the last section summarizes our conclusions.

## 2. Results and Discussion

For pesticides, LC–MS–MS retention times *R*_t_ were taken from Wang and Telepchak. Methamidophos was taken as the reference *R*_t_ (*R*_t_°) because of its least *R*_t_ (*cf*. Table 1). Internal standard (IS) triphenyl phosphate (TPP) was included in the classification. The (*R*_t_–*R*_t_°)/*R*_t_° ratios were calculated. Molecular fractal dimensions were computed with our program TOPO [37].

Variations of (*R*_t_ − *R*_t_°)/*R*_t_° *vs.* 1-octanol–water partition coefficient and fractal dimension averaged for nonburied atoms minus molecular fractal dimension *D’*–*D* show fit. The regression turns out to be:

(*R_t_−R_t_°)/R_t_°*= − 0.188 + 0.367log*P* + 19.6(*D’−D*), *n* = 9, *r* = 0.973, *s* = 0.337, *F* = 53.3
MAPE = 8.77% AEV = 0.0533 (1)

where mean absolute percentage error (MAPE) is 8.77% and approximation error variance (AEV), 0.0533. If IS TPP is excluded the results are improved:

(*R_t_−R_t_°)/R_t_°*= − 0.299 + 0.420log*P* + 19.1(*D’−D*), *n* = 8, *r* = 0.982, *s* = 0.295, *F* = 67.5
MAPE = 7.17% AEV = 0.0357 (2)

and AEV decays by 33%. When *D’* is included in the fit the correlation is bettered:

(*R_t_−R_t_°*)/*R_t_°*= − 11.6 + 0.272log*P* + 9.44*D’*, *n* = 8, *r* = 0.987, *s* = 0.253, *F* = 93.2
MAPE = 5.81% AEV = 0.0261 (3)

and AEV drops by 51%. The best quadratic model *vs. D’* improves the fit:

(*R_t_−R_t_°)/R_t_°*= − 112 + 151 *D’*−49.3 *D’*_2_), *n* = 9, *r* = 0.988, *s* = 0.224, *F* = 125.2
MAPE = 5.39% AEV = 0.0234
(4)

and AEV decreases by 56%. If IS TPP is excluded the results are bettered:

(*R_t_−R_t_°*)/*R_t_°*= − 107 + 144 *D’*−46.7 *D’*_2_), *n* = 8, *r* = 0.989, *s* = 0.228, *F* = 114.4
 MAPE = 5.84% AEV = 0.0214
(5)

and AEV decays by 60%. Model (3) is linear and expected to perform better than Equations (4) and (5) for extrapolation. However, the latter are nonlinear and could function better than Equation (3) for intrapolation. Additional fitting parameters were tested: absolute/differential formation enthalpies, molecular dipole moment, organic solvent/water partition coefficients, free energies of solvation and water → organic solvent transfer, molecular volume, surface area, globularity, rugosity, hydrophobic, hydrophilic and total solvent accessible surfaces, and numbers of P and total atoms. However, the results do not improve Equations (3)–(5).

**Table 1:** Vector property (cyc_123_, O_0345_, NP, S=, N_13_, Cl_3_), retention, log*P*, p*K*_a_ and dimensions for pesticides.

Compound	*R*_t_ (min)	*R*_t_ − *R*_t_° (min)	(*R*_t_ − *R*_t_°)/*R*_t_°	log*P*	p*K*_a_	D	D’
1. Methamidophos C_2_H_8_NO_2_PS <001010>	2.78	0.00	0.00000	−0.779	−0.58	1.235	1.266
2. Carbendazim C_9_H_9_N_3_O_2_ <100010>	6.48	3.70	1.33094	1.52	5.66	1.284	1.332
3. Thiabendazole C_10_H_7_N_3_S <110010>	6.91	4.13	1.48561	2.47	3.40	1.288	1.331
4. Pyrimethanil C_12_H_13_N_3_ <110010>	10.43	7.65	2.75180	2.558	4.41	1.314	1.407
5. Cyprodinil C_14_H_15_N_3_ <110010>	11.44	8.66	3.11511	3.012	4.22	1.344	1.470
6. TPP (IS) C_18_H_15_O_4_P <111000>	11.78	9.00	3.23741	4.63	−5	1.394	1.504
7. Diazinone C_12_H_21_N_2_O_3_PS <111100>	11.92	9.14	3.28777	3.766	1.21	1.398	1.509
8. Pyrazophos C_14_H_20_N_3_O_5_PS <111110>	12.24	9.46	3.40288	2.810	−1.37	1.403	1.505
9. Chlorpyrifos C_9_H_11_NO_3_PSCl_3_ <111111>	13.42	10.64	3.82734	5.004	−5.28	1.394	1.494

Pearson correlation coefficient matrix **R** was calculated between pairs of vector properties <*i*_1_, *i*_2_, *i*_3_,*i*_4_, *i*_5_, *i*_6_> for nine pesticides. Intercorrelations are illustrated in the partial correlation diagram, which contains high (*r* ≥ 0.75), medium (0.50 ≤ *r* < 0.75), low (0.25 ≤ *r* < 0.50) and *zero* (*r* < 0.25) partial autocorrelations. Pairs of molecules with higher partial correlations show similar vector property. However, results should be taken with care, because Entry 9 with constant vector <111111> shows null standard deviation, causing greatest partial correlations *r* = 1 with any compound, which is an artefact. With the equipartition conjecture the upper triangle of **R** resulted:


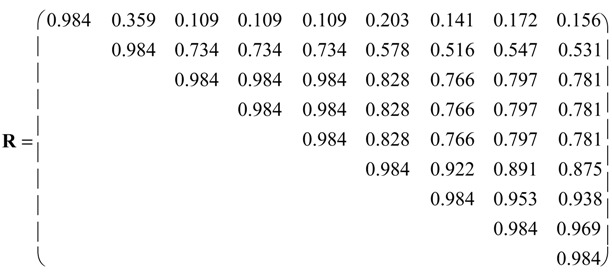


**Figure 1:**
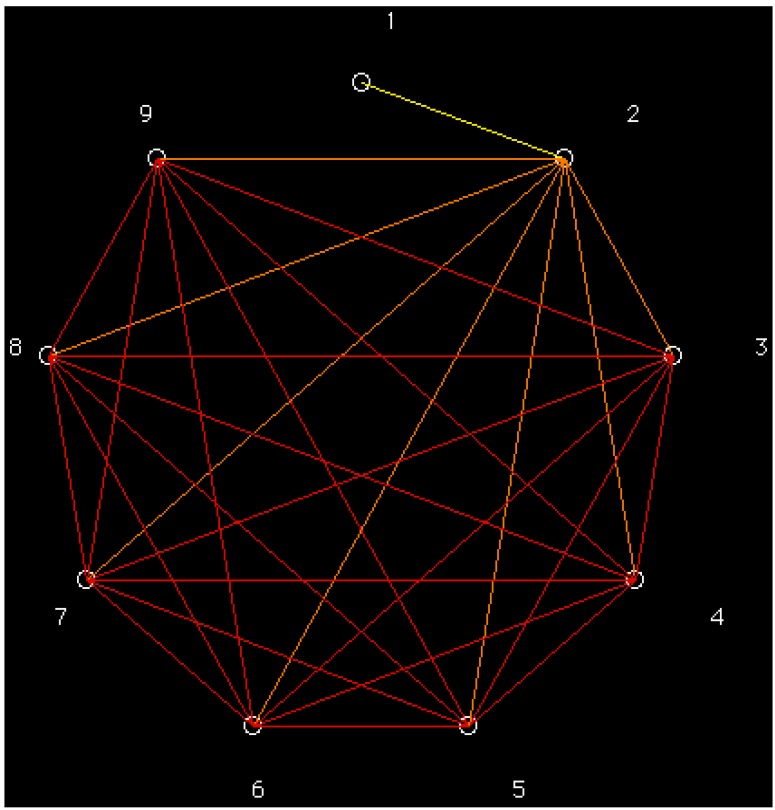
Partial correlation diagram: High (*red*), medium (*orange*) and low (*yellow*) correlations of pesticides.

Some correlations are high, e.g., *R*_3,4_ = *R*_3,5_ = *R*_4,5_ = 0.984. They are illustrated in the partial correlation diagram, which contains 21 high (*cf*. Figure 1, *red lines*), seven medium (*orange*), one low (*yellow*) and seven *zero* (*black*) partial correlations. Two out of eight high partial correlations of Entry 9 are corrected: its correlation with Entry 2 is medium and its correlation with Entry 1 is *zero* partial correlation. For instance, pesticide 2 (carbendazim) shows medium partial correlations with molecules 3–9 (0.50 ≤ *r* < 0.75, *orange*) and low partial correlation with compound 1 (0.25 ≤ *r* < 0.50, *yellow*).

The grouping rule in the case with equal weights *a_k_* = 0.5 for *b*_1_ = 0.93 allows the classes: 

C *−* *b*_1_ = (1)(2)(3,4,5)(6)(7,8,9)

Five clusters are obtained with the associated entropy *h* *–* **R***–* *b*_1_ = 10.70 matching to <*i*_1_,*i*_2_,*i*_3_,*i*_4_,*i*_5_,*i*_6_> and C *−* *b*_1_ [38,39,40]; the binary taxonomy (Table 1) separates the classes 1, 2, 3, 4 and 5 with 1, 1, 3, 1 and 3 pesticides, respectively [41]. The planar molecules 3–5 with low retention are grouped into the same class; nonplanar thiophosphates 7–9 with the greatest retention are aggregated into the same cluster. Substances belonging to the same grouping appear highly correlated in the partial correlation diagram (Figure 1). However, C *–* *b*_1_ results should be taken with care because classes (1), (2) and (6) with only one substance could be outliers. At level *b*_2_ with 0.74 ≤ *b*_2_ ≤ 0.76, the set of groupings turns out to be:

C *− b*_2_ = (1)(2)(3,4,5,6,7,8,9)

Three clusters result and entropy decays to *h –* **R** *– b*_2_ = 3.71 going with <*i*_1_,*i*_2_,*i*_3_,*i*_4_,*i*_5_,*i*_6_> and C *− b*_2_ dividing classes: 1–3 with 1, 1 and 7 pesticides. Again, nonplanar thiophosphates 7–9 with the greatest retention are aggregated into the same class. Compounds in the same cluster appear highly correlated in partial correlation diagram (Figure 1). Notwithstanding, C *– b*_2_ results should be taken with caution because groupings (1) and (2) with a unique compound could be outliers. Table 2 shows comparative analysis of the set containing 1–9 classes in agreement with partial correlation diagram (Figure 1).

From the previous partial correlation diagram (Figure 1) and set of nine classifications (Table 2), we suggest splitting the data into three groupings: 

(1,2)(3,4,5)(6,7,8,9)

**Figure 2:**
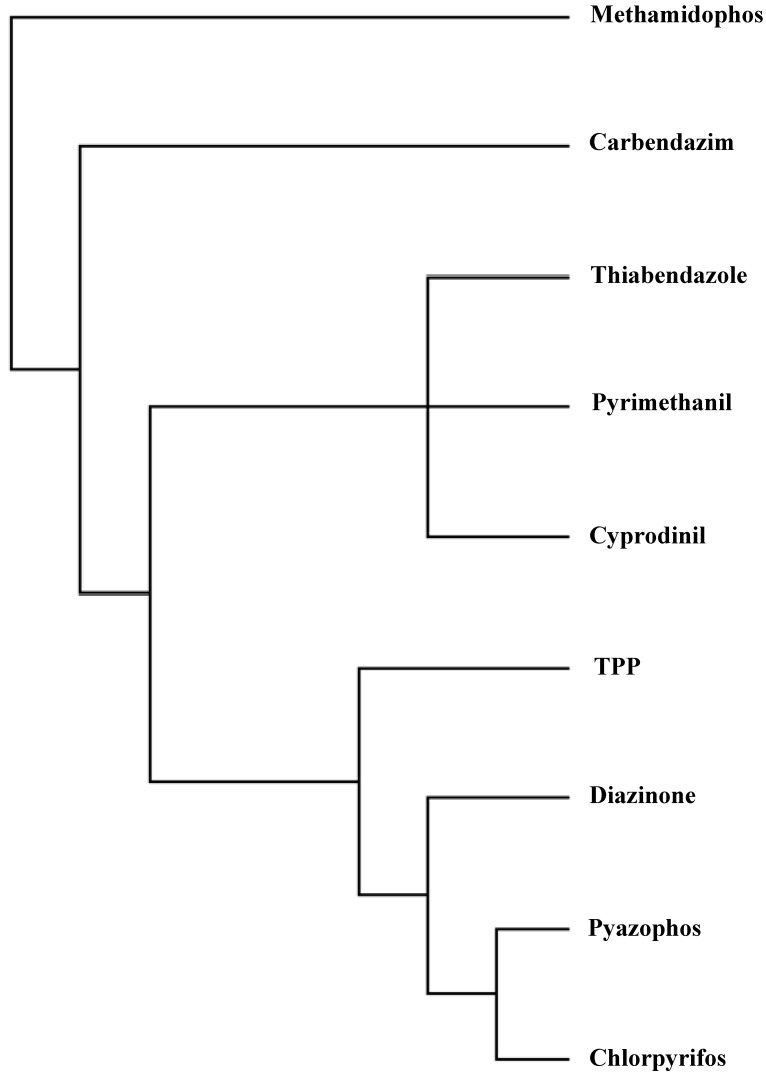
Dendrogram of pesticides according, *top* → *bottom*, to: (1,2)(3,4,5)(6,7,8,9).

The pesticides dendrogram (*cf*. Figure 2) shows different behaviour depending on the number of cyles. One more time, the planar molecules 3–5 with low retention are grouped into the same class and nonplanar thiophosphates 6–9 with the greatest retention are aggregated into the same cluster.

**Table 2:** Classification level, number of classes and entropy for vector property of pesticides.

Classification Level *b*	Number of Classes	Entropy *h*
1.00	9	32.49
0.98	7	20.01
0.96	6	15.13
0.93	5	10.70
0.87	4	6.77
0.76	3	3.71
0.51	2	1.47
0.10	1	0.08

**Figure 3:**
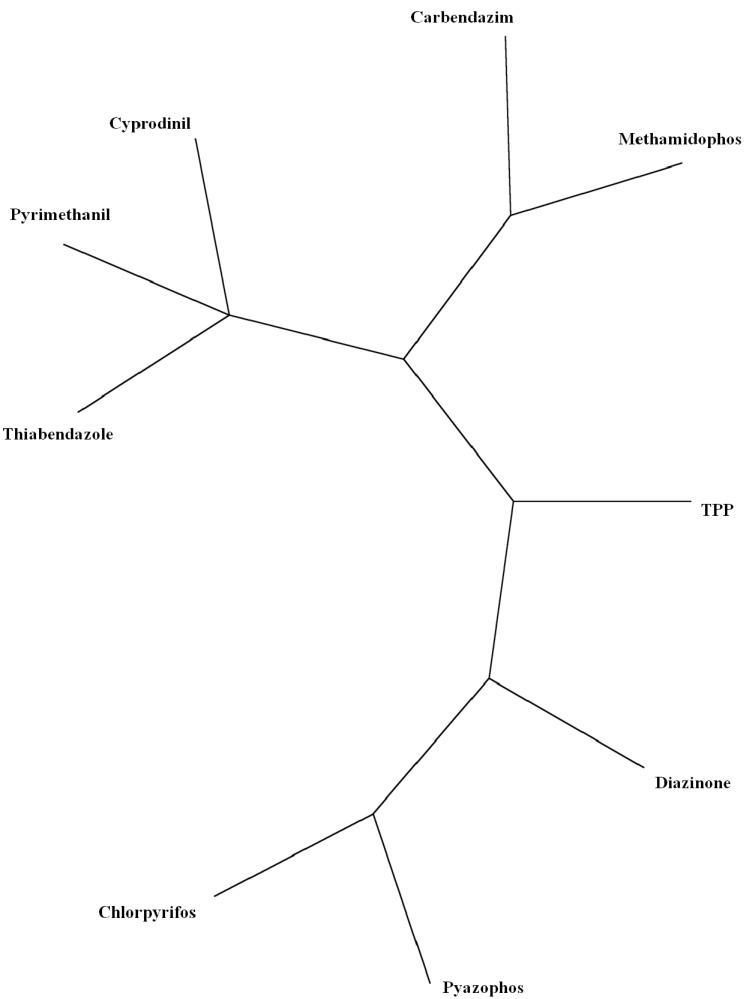
Radial tree of pesticides according, from *top* to *bottom*, to: (1,2)(3,4,5)(6,7,8,9).

The illustration of the classification above in a radial tree (*cf*. Figure 3) shows the different behaviour of the pesticides depending on the number of cyles. The same classes above are recognized, in qualitative agreement with partial correlation diagram and dendrogram (Figure 1 and Figure 2). One more time, planar molecules 3–5 with low retention are grouped into the same class, and nonplanar thiophosphates 6–9 with the greatest retention are aggregated into identical cluster.

**Figure 4:**
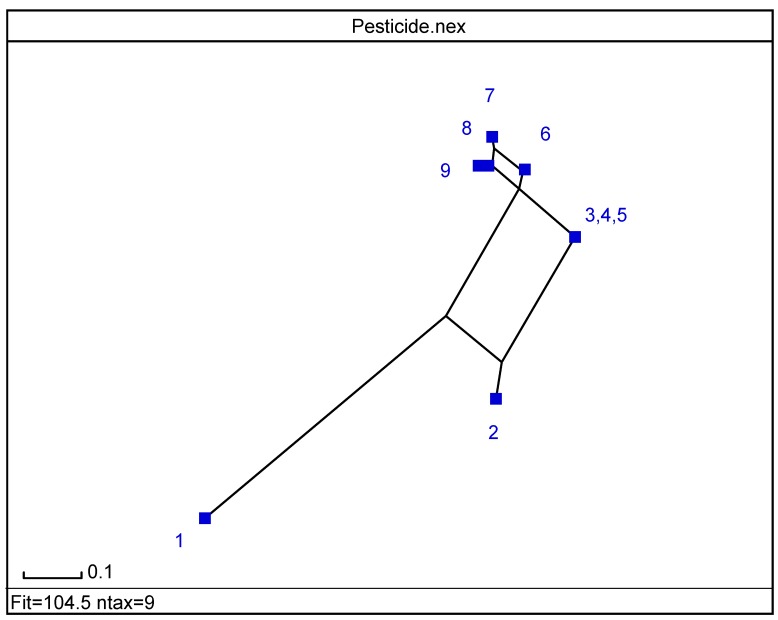
Splits graph of pesticides according, *top* → *bottom*, to: (1,2)(3,4,5)(6,7,8,9).

Program SplitsTree allows examining cluster analysis (CA) data [42]. Based on *split decomposition* it takes as input a *distance matrix* and produces as output a graph, which represents relations between taxa. For ideal data the graph is a tree whereas less ideal data will give rise to a tree-like net, which is interpreted as possible evidence for conflicting data. As split decomposition does not attempt to force data on to a tree it can provide a good indication of how *tree-*like are given data. In the splits graph for nine pesticides (*cf*. Figure 4), points 4 and 5 are superimposed on 3, and 9 on 8. It reveals conflicting relationship between class 1, and groupings 2 and 3 because of interdependences. It indicates spurious relation resulting from base-composition effects. It shows different pesticides behaviour depending on number of cycles in agreement with partial correlation diagram, binary and radial trees (Figure 1,Figure 2 and Figure 3).

In QSPR, the data file contains less than 100 objects and thousands of *X‑*variables. So many *X‑*variables exist that no one can discover by *inspection* patterns, trends, clusters, *etc.* in objects. *Principal components analysis* (PCA) is a technique useful to *summarize* all information contained in **X**‑matrix and put it understandable [43,44,45,46,47,48]. The PCA works decomposing **X**‑matrix as the product of two smaller matrices **P** and **T**. Loading matrix (**P**) with information about the variables contains few vectors, the principal components (PCs), which are obtained as linear combinations of the original *X-*variables. In the score matrix (**T**) with information about objects, every object is described in terms of the projections on to PCs instead of the original variables: **X** = **TP’** + **E**, where **’** denotes the transpose matrix. The information not contained in the matrices remains as *unexplained* X-*variance* in the residual matrix (**E**). Every PC*_i_* is a new co-ordinate expressed as linear combination of old features *x_j_*: PC*_i_* = ∑*_j_b_ij_x_j_*. The new co‑ordinates PC*_i_* are named scores/factors while the coefficients *b_ij_* are called loadings. The scores are ordered according to their information content with regard to the total variance among all objects. *Score–score plots* show positions of compounds in the new co-ordinate system while *loading–loading plots* indicate the locations of the features that represent the compounds in the new co-ordinates. The PCs present two interesting properties. (1) They are extracted in decaying order of importance. First PC *F*_1_ always contains more information than the second *F*_2_ does, *F*_2_ more than the third *F*_3_, *etc.* (2) Every PC is orthogonal to one another. There is no correlation between the information contained in different PCs. A PCA was performed for the vector properties. The importance of PCA factors *F*_1_*–F*_6_ for {*i*_1_,*i*_2_,*i*_3_,*i*_4_,*i*_5_,*i*_6_} is collected in Table 3. The use of the first factor *F*_1_ explains 39% of variance (61% error), combined application of two factors *F*_1/2_ accounts for 66% of variance (34% error), utilization of factors *F*_1–3_ justifies 87% of variance (13% error), *etc.*

**Table 3:** Importance of the principal component analysis factors for vector property of pesticides.

Factor	Eigenvalue	Percentage of Variance	Cumulative Percentage of Variance
*F*_1_	2.33109829	38.85	38.85
*F*_2_	1.62998318	27.17	66.02
*F*_3_	1.25482746	20.91	86.93
*F*_4_	0.38517751	6.42	93.35
*F*_5_	0.33518718	5.59	98.94
*F*_6_	0.06372637	1.06	100.00

The PCA factor loadings are shown in Table 4.

**Table 4:** Principal component analysis loadings for the vector property of pesticides.

Property	PCA Factor Loadings ^a^					
	*F*_1_	*F*_2_	*F*_3_	*F*_4_	*F*_1_	*F*_6_
*i*_1_	0.30822766	0.64474701	0.02673332	−0.04594797	0.50138891	−0.48485077
*i*_2_	0.44804795	0.45046774	−0.05731613	0.08927231	**−0.76002034**	0.08629170
*i*_3_	0.40956062	−0.56947041	−0.18574529	0.04102918	−0.15327503	−0.66954134
*i*_4_	0.55772042	−0.17916516	0.17892539	0.59525799	0.32511366	0.40595876
*i*_5_	−0.31588577	0.04253838	**0.72852310**	0.44514023	−0.20425092	−0.35748077
*i*_6_	0.35450381	−0.15222972	0.63145758	−0.66011661	0.00822715	0.12881324

^a^ Loadings greater than 0.7 are boldfaced.

The PCA *F*_1_*–F*_3_ profile for the vector property is listed in Table 5. For *F*_1_, variable *i*_4_ shows the greatest weight in the profile; however, *F*_1_ cannot be reduced to two variables {*i*_2_,*i*_4_} without 49% error. For *F*_2_, variable *i*_1_ presents the greatest weight; notwithstanding, *F*_2_ cannot be reduced to two variables {*i*_1_,*i*_3_} without 26% error. For *F*_3_, variable *i*_5_ displays the greatest weight; nevertheless, *F*_3_ cannot be reduced to two variables {*i*_5_,*i*_6_} without 7% error. For *F*_4_, variable *i*_6_ exhibits the greatest weight; however, *F*_4_ cannot be reduced to two variables {*i*_4_,*i*_6_} without 21% error. For *F*_5_, variable *i*_2_ reveals the greatest weight; notwithstanding, *F*_5_ cannot be reduced to two variables {*i*_1_,*i*_2_} without 17% error. For *F*_6_, variable *i*_3_ bares the greatest weight; nevertheless, *F*_6_ cannot be reduced to two variables {*i*_1_,*i*_3_} without 32% error. Factors *F*_1–6_ can be considered as the linear combinations of {*i*_2_,*i*_4_}, {*i*_1_,*i*_3_}, {*i*_5_,*i*_6_}, {*i*_4_,*i*_6_}, {*i*_1_,*i*_2_} and {*i*_1_,*i*_3_} with 49%, 26%, 7%, 21%, 17% and 32% errors.

**Table 5:** Profile of the principal component analysis factors for the vector property of pesticides.

	Percentage of i_1_^a^	% of i_2_	% of i_3_	% of i_4_	% of i_5_	% of i_6_
F_1_	9.50	20.07	16.77	31.11	9.98	12.57
F_2_	41.57	20.29	32.43	3.21	0.18	2.32
F_3_	0.07	0.33	3.45	3.20	**53.07**	39.87
F_4_	0.21	0.80	0.17	35.43	19.81	43.58
F_5_	25.14	**57.76**	2.35	10.57	4.17	0.01
F_6_	23.51	0.74	44.83	16.48	12.78	1.66

^a^ Percentages greater than 50% are boldfaced.

**Figure 5:**
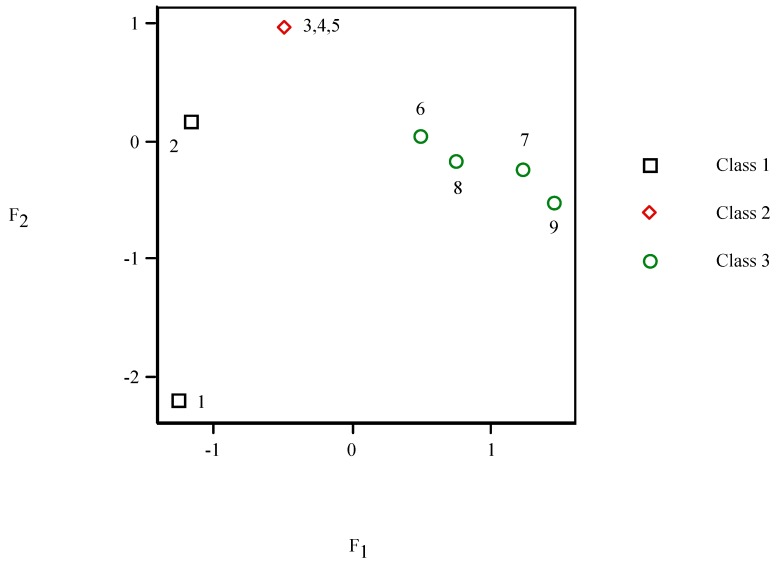
*F*_2_ *versus F*_1_ scores plot of the principal component analysis for the pesticides.

In PCA *F*_2_*–F*_1_ scores plot (*cf*. Figure 5), points 4 and 5 appear superimposed on 3. It shows different behaviour depending on number of cyles. It distinguishes three clusters: class 1 (two molecules, *F*_1_ < *F*_2_, *left*), grouping 2 (three compounds, *F*_1_ << *F*_2_, *top*) and cluster 3 (four units, *F*_1_ >> *F*_2_, *right*).

**Figure 6:**
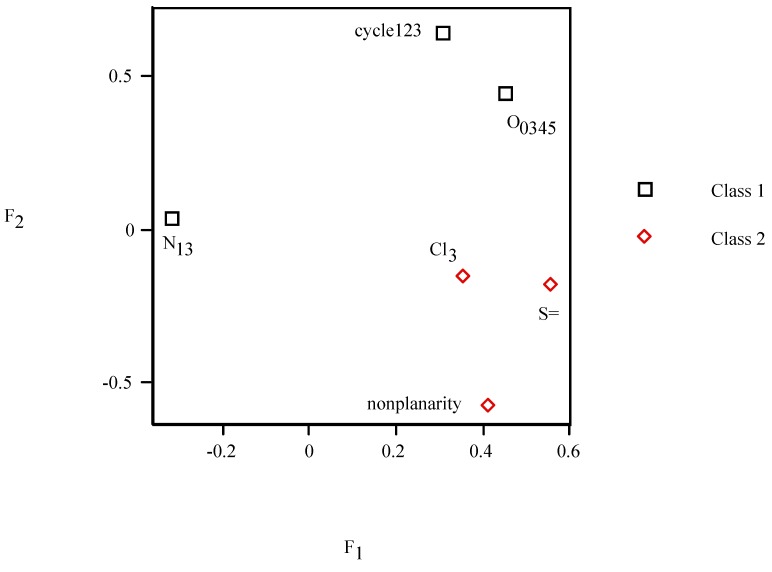
*F*_2_ *versus F*_1_ loadings plot of the principal component analysis for the pesticides.

From PCA factor loadings of pesticides, *F*_2_*–F*_1_ loadings plot (*cf*. Figure 6) depicts the six properties. In addition as a complement to the scores plot (Figure 5) for the loadings (Figure 6), it is confirmed that pesticide 2 located on the left side presents a contribution of cyc_123_ situated near the same side of Figure 5. Class 2 on the top shows more pronounced contribution of O_0345_ placed in the same position (Figure 6). Two classes of properties are clearly distinguished in the loadings plot: class 1 {cyc_123_,O_0345_,N_13_} (*F*_1_ < *F*_2_, *top*) and grouping 2 {NP,S=,Cl_3_} (*F*_1_ >> *F*_2_, *bottom*).

Instead of nine pesticides in the ℜ^6^ space of six vector properties, consider six properties in the ℜ^9^ space of nine molecules. The upper triangle of matrix **R** between pairs of properties resulted in:


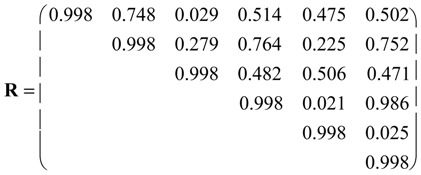


**Figure 7:**
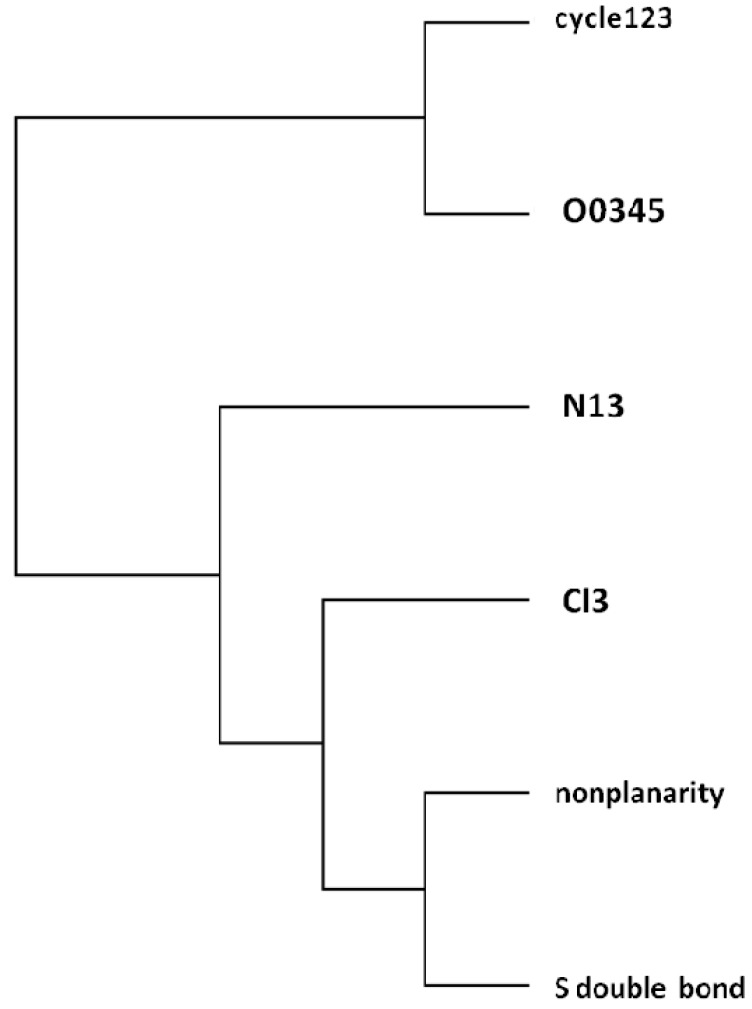
Dendrogram for the vector properties corresponding to the pesticides.

Correlations are high, e.g., *R*_4,6_ = 0.986. Properties dendrogram (*cf*. Figure 7) separates cyc_123_ and O_0345_ from N_13_ (class 1), and Cl_3_ from NP/S= (cluster 2) in agreement with PCA loadings plot (Figure 6).

**Figure 8:**
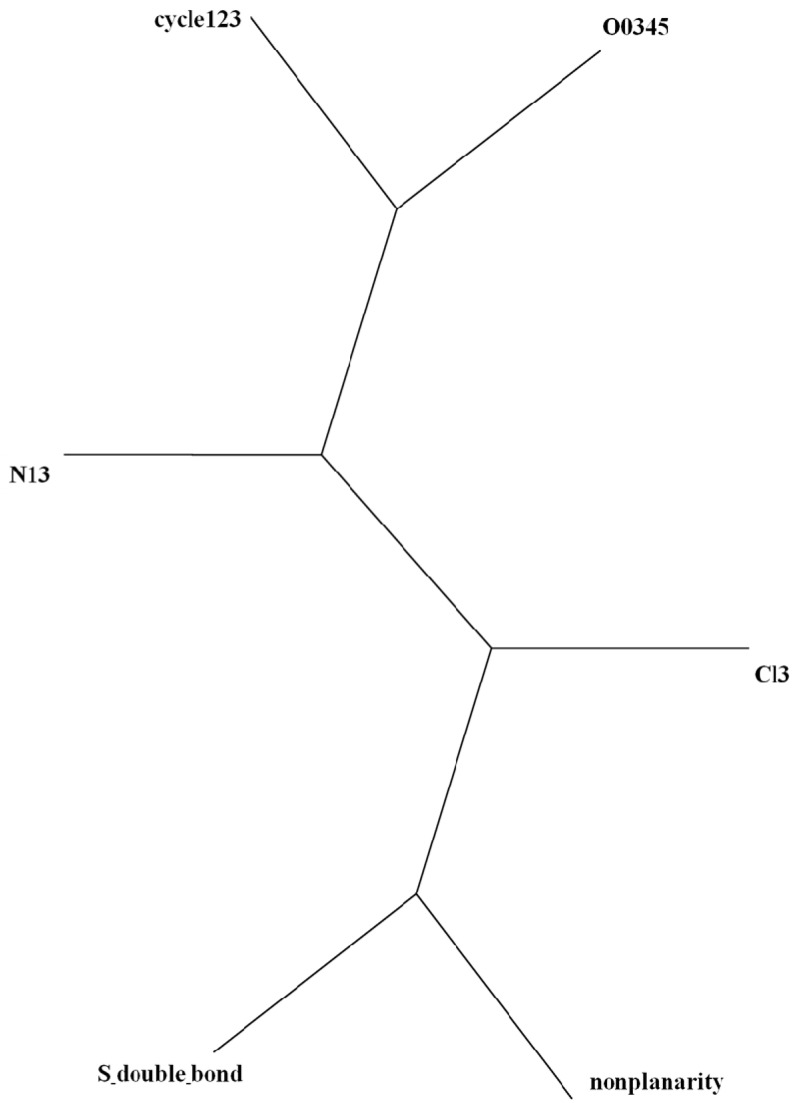
Radial tree for the vector properties corresponding to the pesticides.

**Figure 9:**
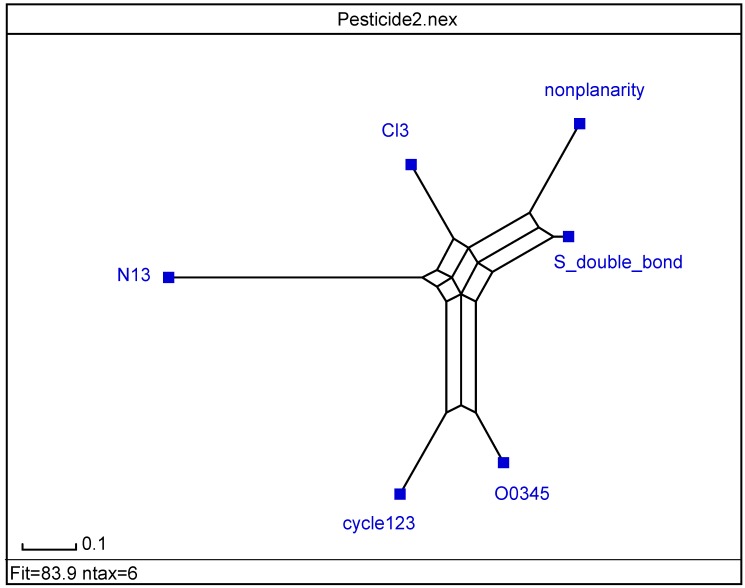
Splits graph for the vector properties corresponding to the pesticides.

The radial tree for the vector properties (*cf*. Figure 8) separates the same two classes as PCA loadings plot and dendrogram (Figure 6 and Figure 7). Splits graph for properties (*cf*. Figure 9) reveals conflicting relation between classes because of interdependences. It is in agreement with PCA loadings plot and binary/radial trees (Figure 6, Figure 7 and Figure 8).

**Figure 10:**
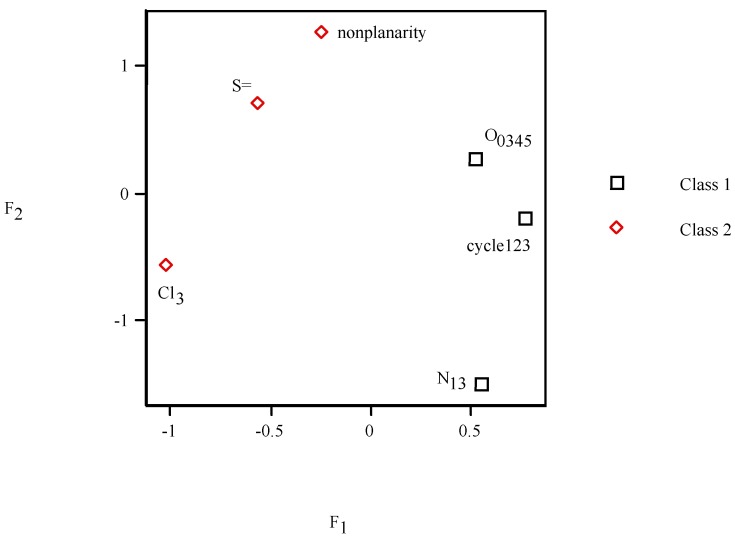
PCA *F*_2_ *vs. F*_1_ scores plot for the vector properties corresponding to pesticides.

A PCA was performed for the vector properties. Factor *F*_1_ explains 50% of variance (50% error), factors *F*_1/2_ account for 69% of variance (31% error), factors *F*_1–3_ rationalize 82% of variance (18% error), *etc.* In PCA *F*_2_*–F*_1_ scores plot, the same two groupings of properties are distinguished: class 1 {cyc_123_,O_0345_,N_13_} (*F*_1_ >> *F*_2_, *cf*. Figure 10, *right*) and grouping 2 {NP,S=,Cl_3_} (*F*_1_ << *F*_2_, *left*) in qualitative agreement with PCA loadings plot, binary/radial trees and splits graph (Figure 6, Figure 7, Figure 8 and Figure 9).

The recommended format of the pesticides periodic table (PT, *cf*. Table 6) shows that they are classified first by *i*_1_, *i*_2_, *i*_3_, *i*_4_, *i*_5_ and, finally, by *i*_6_. Vertical groups are defined by <*i*_1_,*i*_2_,*i*_3_,*i*_4_,*i*_5_> and horizontal periods, by <*i*_6_>. Periods of eight units are assumed; e.g., group g00101 stands for <*i*_1_,*i*_2_,*i*_3_,*i*_4_,*i*_5_> = <00101>: <001010> (cyc_0_,O_2_,NP,S=_0_,N_1_,Cl_0_), *etc.* Pesticides in the same column appear close in partial correlation diagram, binary/radial trees, splits graph and PCA scores (Figure 1, Figure 2, Figure 3, Figure 4 and Figure 5). Phenylurea herbicides were determined in tap water/soft drink samples by HPLC–UV [49]. Table 6 includes five phenylureas: metazachlor is similar to carbendazim. Can *et al.* determined sulphonyl/phenylurea herbicides toxicities [50]. Table 6 includes 27 sulphonyl/phenylurea herbicides: (1) phenylureas are similar to metoxuron, monuron, diuron and linuron; (2) sulphonylureas flazasulphuron, triasulphuron, azimsulphuron and chlorsulphuron go with TPP. High-resolution and ultratrace analyses of pesticides were reported via silica (SiO_2_) monoliths [51]. Table 6 takes in six new pesticides: (1) metamitron and phenylurea isoproturon match metoxuron; (2) metolachlor goes with carbendazim; (3) carbofuran agrees with thiabendazole. Qualitative LC–MS analysis of pesticides was informed via monolithic SiO_2_ capillaries [52]. Table 6 contains two novel pesticides: phenylurea pencycuron tallies metamitron. Analytical standards were provided for persistent organic pollutants (POPs) [53]. Table 6 embraces five POPs: lindane and pentachlorobenzene equal carbetamide

**Figure 11:**
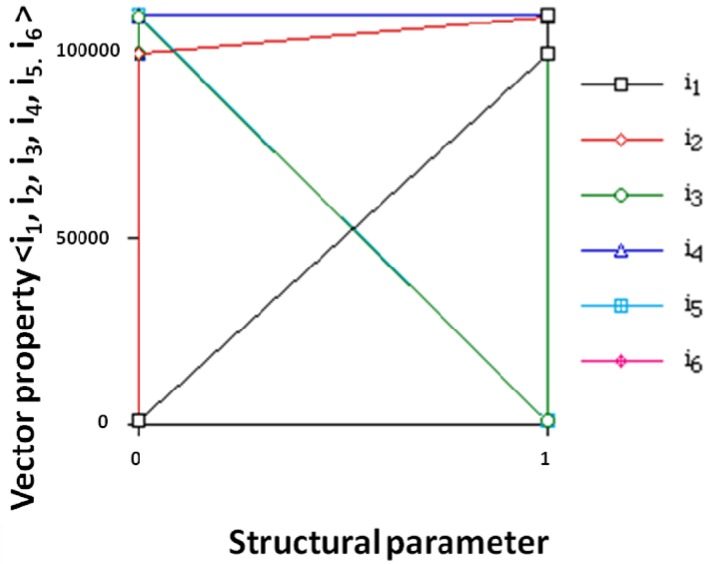
Variation of vector property *P*(*p*) of the pesticides *versus* counts of {*i*_1_,*i*_2_,*i*_3_}.

Property *P* variation of vector <*i*_1_,*i*_2_,*i*_3_,*i*_4_,*i*_5_,*i*_6_> (*cf*. Figure 11) is expressed in decimal system, *P* = 10^5^*i*_1_ + 10^4^*i*_2_ + 10^3^*i*_3_ + 10^2^*i*_4_ + 10*i*_5_ + *i*_6_, *vs.* structural parameters {*i*_1_,*i*_2_,*i*_3_,*i*_4_,*i*_5_,*i*_6_} for the pesticides. Most points and lines *i*_3_/*i*_5_ collapse. For instance, for molecule 1 (methamidophos) <001010>, vector property *P* = 10^5^·0 + 10^4^·0 + 10^3^·1 + 10^2^·0 + 10·1 + 0 = 1010 where the structural parameters are 0 and 1, and the corresponding points are (*i*_1_ = *i*_2_ = *i*_4_ = *i*_6_ = 0, *P* = 1010) and (*i*_3_ = *i*_5_ = 1, *P* = 1010). The results show parameters hierarchy: *i*_1_ > *i*_2_ > *i*_3_ > *i*_4_ > *i*_5_ > *i*_6_ in agreement with PT of properties (Table 6) with vertical groups defined by {*i*_1_,*i*_2_,*i*_3_,*i*_4_,*i*_5_} and horizontal periods described by {*i*_6_}. The property was not used in PT development and validates it.

**Table 6:** Table of periodic properties for pesticides, persistent organic pollutants, phenylureas and sulphonylureas.

P.	g00100	g00101	g01100	g10000	g10001	g10100	g11000	g11001	g11100	g11110	g11111
p0	Chlordecone**	Methamidophos	PFOS**	Metamitron* BDE‑99** *Metoxuron* ***Monuron*** ***Diuron*** ***Linuron*** ***Buturon*** ***Chlorotoluron*** ***Daimuron*** ***Fenuron*** ***Methyldimuron*** ***Fluometuron*** ***Siduron*** ***Neburon*** ***Isoproturon*** ***Pencycuron***	Carbendazim Metolachlor* ***Metazachlor***	**AMS** **BSM** **CME** **CNS** **EMS** **MSM** **NCS** **OXS** **PSE** **TFS** **TBM** **TFO** **3FS** **RMS** IDS	Carbetamide* Prometryne* Lindane** PCB**	Thiabendazole Pyrimethanil Cyprodinil Carbofuran*	TPP **Flazasulphuron** **Triasulphuron** **Azimsulphuron** **Chlorsulphuron**	Diazinone	Pyrazophos
p1									Chlorfenvinphos**		Chlorpyrifos

PFOS: perfluoroctane sulphonate. BDE‑99: 2,2',4,4',5‑pentabromodiphenylether. AMS: amidosulphuron. BSM: bensulphuron‑methyl. CME: Chlorimuron‑ethyl. CNS: Cinosulphuron. EMS: ethametsulphuron‑methyl. MSM: metsulphuron‑methyl. NCS: nicosulphuron. OXS: oxasulphuron. PSE: pyrazosulphuron‑ethyl. TFS: Thifensulphuron‑methyl. TBM: Tribenuron‑methyl. TFO: Trifloxysulphuron‑Na. 3FS: triflusulphuron‑methyl. RMS: rimsulphuron. IDS: iodosulphuron. PCB: pentachlorobenzene. Regular typeface: pesticides (this work). Regular typeface *: pesticides taken from Ref. [51]. Regular typeface **: persistent organic pollutants. *Italics*: phenylureas. **Bold:** sulphonylureas.

**Figure 12:**
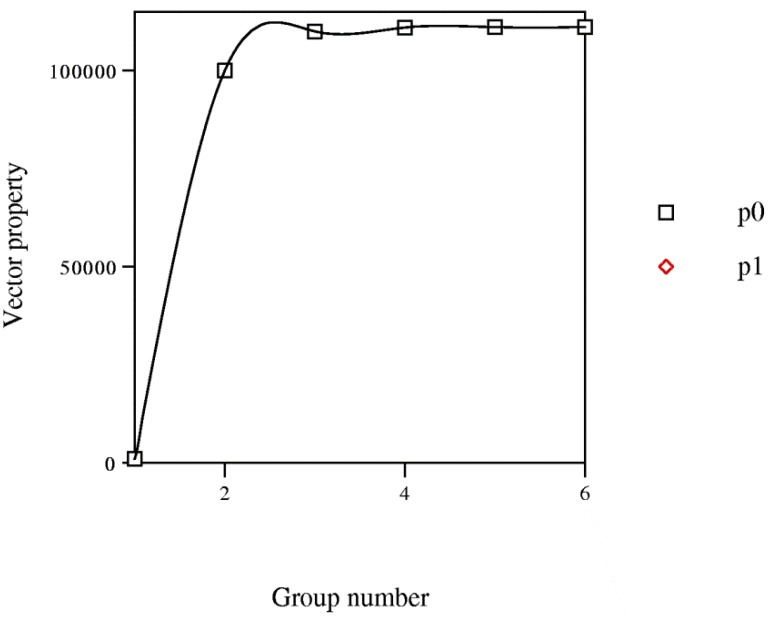
Variation of the vector property *P*(*p*) of the pesticides *versus* group number.

Property *P* change of vector <*i*_1_,*i*_2_,*i*_3_,*i*_4_,*i*_5_,*i*_6_> in base 10 (*cf*. Figure 12) is represented *vs.* number of group in PT, for pesticides (Table 1 subset of Table 6). It reveals minima corresponding to compounds with <*i*_1_,*i*_2_,*i*_3_,*i*_4_,*i*_5_> *ca*. <00101> (group g00101) and maxima with <*i*_1_,*i*_2_,*i*_3_,*i*_4_,*i*_5_> *ca*. <11111> (group g11111). For group 6, period 2 is superimposed on 1. For instance, for group g001010 and period p0, molecule 1 (methamidophos) <001010> lies in the first group in the subset with *P* = 1010 and the point is (group = 1, *P* = 1010). Periods p0 and p1 represent rows 1 and 2, respectively, in Table 6. Function *P*(*i*_1_,*i*_2_,*i*_3_,*i*_4_,*i*_5_,*i*_6_) denotes two periodic *waves* clearly limited by two maxima, which suggest a periodic behaviour that recalls form of a trigonometric function. For <*i*_1_,*i*_2_,*i*_3_,*i*_4_,*i*_5_,*i*_6_>, a maximum is shown. Distance in <*i*_1_,*i*_2_,*i*_3_,*i*_4_,*i*_5_,*i*_6_> units between each pair of consecutive maxima is six, which coincides with pesticide sets in successive periods. The maxima occupy analogous positions and are in phase. The representative points in phase should correspond to elements in the same group in PT. For both maxima, <*i*_1_,*i*_2_,*i*_3_,*i*_4_,*i*_5_,*i*_6_> some coherence exists between two representations; however, the consistency is not general. Waves comparison shows two differences: period 1 is somewhat step-like and period 2 is incomplete. The most characteristic points are maxima, which lie about group g11111. The values of <*i*_1_,*i*_2_,*i*_3_,*i*_4_,*i*_5_,*i*_6_> are repeated as the periodic law (PL) states.

An empirical function *P*(*p*) reproduces the different <*i*_1_,*i*_2_,*i*_3_,*i*_4_,*i*_5_,*i*_6_> values. The minimum of *P*(*p*) has meaning only if it is compared with former *P*(*p* – 1) and later *P*(*p* + 1) points needing to fulfill:

*P*_min_ (*P*) < *P*(*P*−1) *P*_min_ (*P*) < *P*(*P*+1) (6)

The order relations (6) should repeat at determined intervals equal to period size and are equivalent to:

*P*_min_ (*P*) − *P*(*P* − 1) <0 *P*(*P*+1) − *P*_min_ (*P*) >0 (7)

As relations (7) are valid only for minima, more general ones are desired for all values of *p*. Differences *D*(*p*) = *P*(*p* + 1) − *P*(*p*) are calculated assigning every value to pesticide *p*:

*D*(*P*) = *P*(*P*+1) − *P*(*P*) (8)

Instead of *D*(*p*), *R*(*p*) = *P*(*p* + 1)/*P*(*p*) is taken, assigning them to pesticide *p*. If PL were general, elements in the same group in analogous positions in different periodic waves would satisfy:

either *D*(*P*)>0 or *D*(*P*)<0 (9)

and either *R*(*P*) >1 or *R*(*P*) <1 (10)

**Figure 13:**
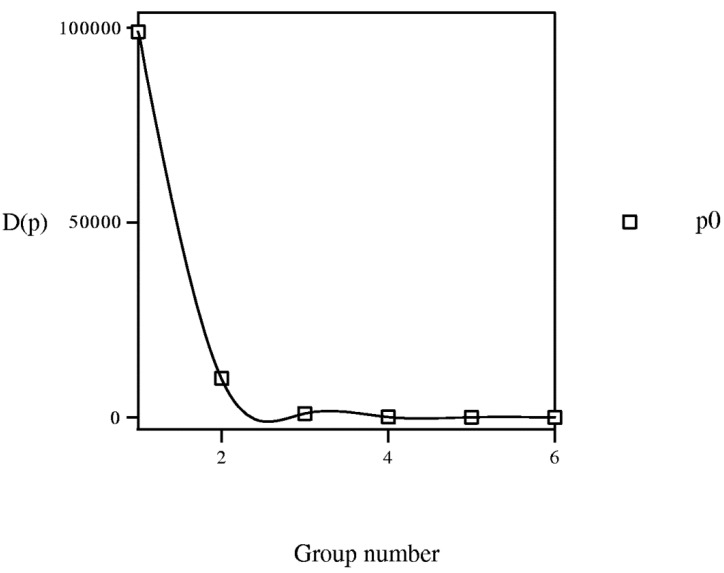
Variation of property *D*(*p*) = *P*(*p* + 1) − *P*(*p*) *versus* group. *P*: vector property.

However, the results show that this is not the case so that PL is not general, existing some anomalies; e.g., *D*(*p*) variation *vs.* group number (*cf*. Figure 13) presents lack of coherence between <*i*_1_,*i*_2_,*i*_3_,*i*_4_,*i*_5_,*i*_6_> Cartesian and PT representations. For instance, for group g001010 and period p0, pesticide 1 (methamidophos) <001010> (group = 1, *P* = 1010) presents, in the next PT position, molecule 2 (carbendazim) <100010> (g100010, group = 2, *P* = 100010), *D* = 100010 − 1010 = 99000 and the point is (group = 1, *D* = 99000). If consistency were rigorous, all points in each period would have the same sign. In general, a trend exists in points to give *D*(*p*) > 0, especially for lower groups.

**Figure 14:**
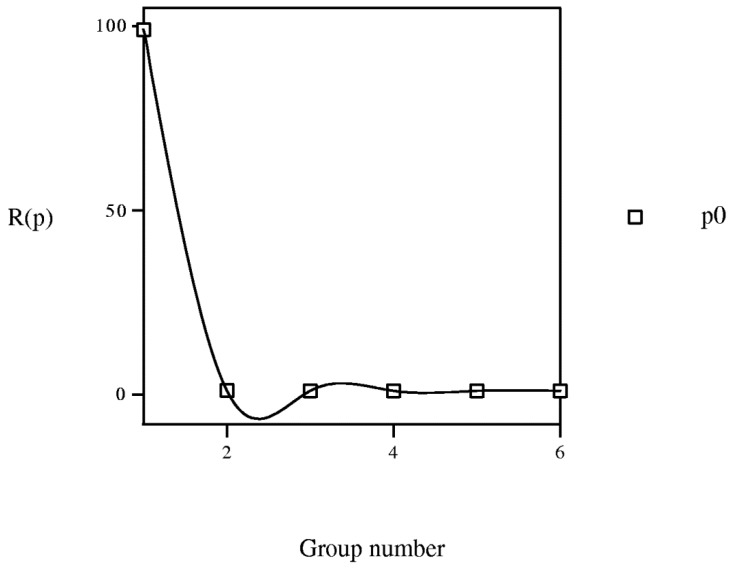
Variation of property *R*(*p*) = *P*(*p* + 1)/*P*(*p*) *vs.* group number. *P*: vector property.

The change of *R*(*p*) *vs.* group number (*cf*. Figure 14) confirms the lack of constancy between Cartesian and PT charts. For instance, for group g001010 and period p0, pesticide 1 (methamidophos) <001010> (group = 1, *P* = 1010) shows, in the next PT cell, molecule 2 (carbendazim) <100010> (g100010, group = 2, *P* = 100010), *R* = 100010/1010 = 99.0198 and the point is (group = 1, *R* = 99.0198). If the steadiness were exact, all points in each period would show *R*(*p*) either lesser or greater than one. A trend exists to give *R*(*p*) > 1, especially for the lower groups.

## 3. Experimental

**Figure 15:**
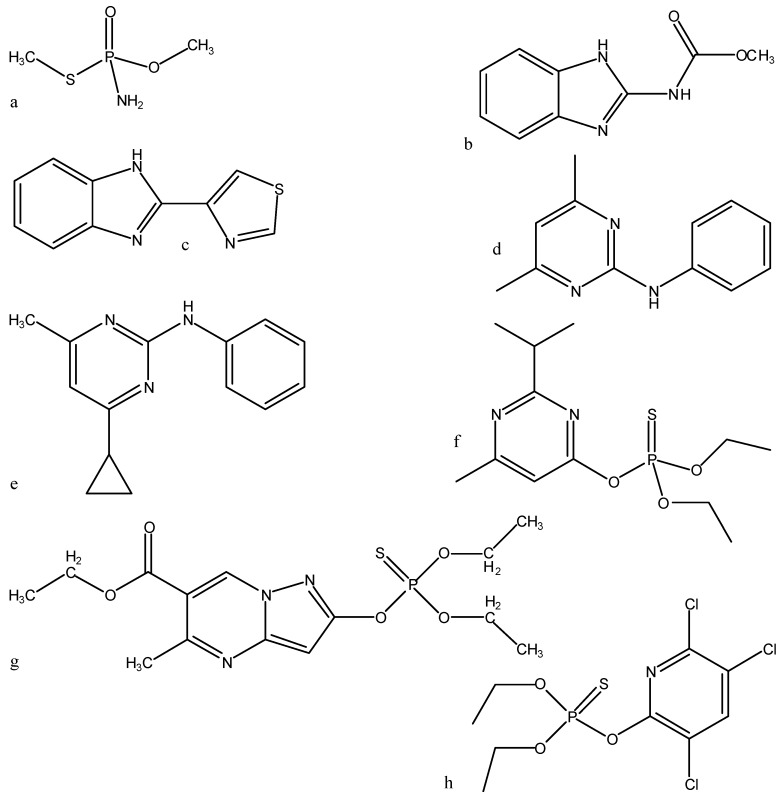
Pesticides: (**a**) methamidophos, (**b**) carbendazim, (**c**) thiabendazole, (**d**) pyrimethanil, (**e**) cyprodinil, (**f**) diazinone, (**g**) pyrazophos and (**h**) chlorpyrifos.

The key problem in classification studies is to define *similarity indices* when several criteria of comparison are involved. The first step in quantifying similarity concept for pesticides is to list the most important chemical characteristics of molecules. The *vector of properties* 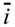 = <*i*_1_,*i*_2_,…*i_k_*,…> should be associated with every pesticide *i*, whose components correspond to different molecular features in a hierarchical order according to their expected importance in retention. If characteristic *m-th* is chromatographically more significant for retention than *k-th* then *m* < *k*. Components *i_k_* are either “1” or “0”, according to whether a similar characteristic of rank *k* is either present or absent in pesticide *i* compared to a reference. Analysis includes six structural and constitutional characteristics: presence of cycle (cyc_123_), occurrence of either none or 3–5 O atoms (O_0345_), nonplanarity (NP), double-bonded S atom (S=), incidence of either one or three N atoms (N_13_) and existence of three Cl atoms (Cl_3_, *cf*. Figure 15). It is assumed that the *chemical characteristics* can be *ranked* according to their contribution to retention in the following order of decaying importance: cyc_123_ > O_0345_ > NP > S= > N_13_ > Cl_3_. Index *i*_1_ = 1 denotes cyc_123_ (*i*_1_ = 0 for cyc_0_), *i*_2_ = 1 means O_0345_ (*i*_2_ = 0 for O_2_), *i*_3_ = 1 signifies NP, *i*_4_ = 1 indicates S=, *i*_5_ = 1 stands for N_13_ (*i*_5_ = 0 for N_0_ or N_2_) and *i*_6_ = 1 represents Cl_3_ (*i*_6_ = 0 for Cl_0_). In chlorpyrifos number of cycles is one, O is three, it is NP and S=, number of N is one and number of Cl atoms is three; obviously its associated vector is <111111>. In this study chlorpyrifos was selected as *reference* because of its greatest retention. Table 1 contains vectors associated with nine pesticides. Vector <001010> is associated with methamidophos since it shows cyc_0_, O_2_, NP, not S=, N_1_ and Cl_0_.

Let us denote by *r_ij_*(0 ≤ *r_ij_*≤ 1) similarity index of two pesticides associated with vectors and , respectively. Similitude relation is characterized by *similarity matrix***R** = [*r_ij_*]. Similarity index between two pesticides 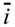 = <*i*_1_,*i*_2_,…*i_k_*…> and 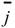 = <*j*_1_,*j*_2_,…*j_k_*…> is defined as:

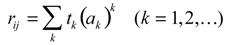
(11)
where 0 ≤ *a_k_* ≤ 1 and *t_k_* = 1 if *i_k_* = *j_k_* but *t_k_* = 0 if *i_k_* ≠ *j_k_*. Definition assigns a weight (*a_k_*)*^k^* to any property involved in description of molecule *i* or *j*.

### 3.1. Classification Algorithm

*Grouping algorithm* uses the *stabilized* similarity matrix by applying *max–min composition rule o*:

(**R**o**S**)*_ij_* = max*_k_* [min*_k_*(*r_ik_*,*s_kj_*)] (12)

where **R** = [*r_ij_*] and **S** = [*s_ij_*] are matrices of equal type and (**R**o**S**)*_ij_* is the (*i*,*j*)*‑th* element of matrix **R**o**S**[54,55,56,57]. When applying composition rule max–min iteratively so that **R**(*n* + 1) = **R**(*n*) o **R**, an integer *n* exists such that: **R**(*n*) = **R**(*n* + 1) = … Matrix **R**(*n*) is called *stabilized similarity matrix*. Its importance lies in fact that in classification it generates partition into disjoint classes. Stabilized matrix is designated by **R**(*n*) = [*r_ij_*(*n*)]. *Grouping rule* follows: *i* and *j* are assigned to the same class if *r_ij_*(*n*) ≥ *b*. Class of *i* noted 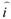 is set of species *j* that satisfies rule: *r_ij_*(*n*) ≥ *b*. Matrix of classes is:

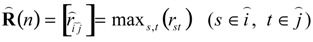
(13)
where *s* stands for any index of species belonging to class 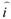
(similarly for *t* and 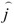). Rule (13) means finding largest similarity index between species of two different classes.

### 3.2. Information Entropy

In information theory, *information entropy h* measures the surprise that source emitting sequences, e.g., cannon-shots, can give [58,59]. Consider use of qualitative spot test to determine the presence of Fe in a water sample. Without any history of testing the analyst must begin by assuming that the two outcomes 0/1 (Fe absent/present) are equiprobable with probabilities 1/2. When up to two metals may be present in sample, e.g., Fe or Ni, four possible outcomes exist, ranging from neither (0,0) to both present (1,1) with equal probabilities 1/4. Which of four possibilities turns up can be determined via two tests each having two observable states. Similarly with three elements eight possibilities exist each with probability of 1/8 = 1/2^3^; three tests are needed. Pattern relates uncertainty and information needed to resolve it. Number of possibilities is expressed to power of 2. Power to which 2 must be raised to give number of possibilities *N* is defined as logarithm to base 2 of that number. Information/uncertainty can be defined in terms of logarithm to base 2 of number of possible analytical outcomes: *I* = *H* = log_2_ *N* = log_2_ 1/*p* = –log_2_ *p*, where *I* is information contained in answer given that *N* possibilities existed, *H*, initial uncertainty resulting from need to consider *N* possibilities and *p*, probability of each outcome if all *N* possibilities are equally likely to occur. The expression is generalized to a situation in which the probability of every outcome is unequal. If one knows from past experience that some elements are more likely to be present than others, the equation is adjusted so that logarithms of individual probabilities suitable weighted are summed: *H* = – Σ *p_i_* log_2_ *p_i_*, where Σ *p_i_* = 1. Consider original example except that now past experience showed that 90% of samples contained no Fe. Degree of uncertainty is calculated using: *H* = –(0.9 log_2_ 0.9 + 0.1 log_2_ 0.1) = 0.469 bits. For a single event occurring with probability *p* degree of surprise is proportional to −ln *p*. Generalizing result to random variable *X* (which can take *N* possible values *x*_1_, …, *x_N_* with probabilities *p*_1_, …, *p_N_*) average surprise received on learning *X* value is: – Σ *p_i_* ln *p_i_*. Information entropy associated with similarity matrix **R** is:

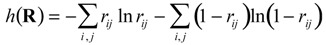
(14)


Denote by *C_b_* set of classes and by 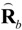 similarity matrix at grouping level *b*. Information entropy satisfies following properties. (1) *h*(**R**) = 0 if either *r_ij_* = 0 or *r_ij_* = 1; (2) *h*(**R**) is maximum if *r_ij_* = 0.5,*i.e.*, when imprecision is maximum; (3) h(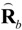)≤*h*(**R**) for any *b*, *i.e.*, classification leads to entropy loss; (4) h
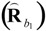
≤*h*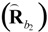 if *b*_1_ < *b*_2_, *i.e.*, entropy is monotone function of grouping level *b*.

### 3.3. Equipartition Conjecture of Entropy Production

In classification algorithm every *hierarchical tree* corresponds to entropy dependence on grouping level and diagram *h − b* is obtained. Tondeur and Kvaalen *equipartition conjecture of entropy production* is proposed as selection criterion among hierarchical trees. According to conjecture for given charge, *dendrogram* (binary tree) with best configuration is that in which entropy production is most uniformly distributed. One proceeds by analogy using *information* instead of thermodynamic one. Equipartition implies linear dependence so that *equipartition line* results:

*h*_eqp_ = *h*_max_*b* (15)

Since classification is discrete, way of expressing equipartition would be regular staircase function. Best variant is chosen to be that minimizing sum of squares of deviations:

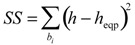
(16)


### 3.4. Learning Procedure

*Learning procedures* were implemented similar to those encountered in *stochastic methods*[60]. Consider a given partition into classes as *good* from practical observations, which corresponds to *reference* similarity matrix **S** = [*s_ij_*] obtained for equal weights *a*_1_ = *a*_2_ = … = *a* and for an arbitrary number of fictious properties. Next consider the same set of species as in the good classification and actual properties. Degree of similarity *r_ij_* is computed with Equation (11) giving matrix **R**. Number of properties for **R** and **S** differs. Learning procedure consists in finding classification results for **R** as close as possible to *good* classification. First weight *a*_1_ is taken constant and only following weights *a*_2_, *a*_3_,… are subjected to random variations. New similarity matrix is obtained using Equation (11) and new weights. Distance between partitions into classes characterized by **R** and **S** is:

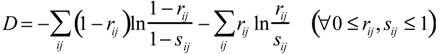
(17)

Definition was suggested by that introduced in information theory to measure distance between two probability distributions [61]. In the present case it is distance between matrices **R** and **S**. Since for every matrix a corresponding classification exists two classifications will be compared by distance, which is nonnegative quantity that approaches zero as resemblance between **R** and **S** increases. The algorithm result is a set of weights allowing classification. The procedure was applied to the synthesis of complex dendrograms using information entropy [62,63,64,65]. Our program MolClas is simple, reliable, efficient and fast procedure for molecular classification, based on equipartition conjecture of entropy production according to Equations (11)–(17); it reads number of properties and molecular properties; it allows optimization of coefficients; it optionally reads starting coefficients and number of iteration cycles. Correlation matrix can be either calculated by program or read from input file. The MolClas calculates property similarity matrix in symmetric storage mode; it applies graphical correlation model for partial correlation diagram; it computes classifications, calculates distances between clusters, computes groupings similarity matrices, works out classifications information entropy, optimizes coefficients, performs single/complete-linkage hierarchical cluster analyses and plots cluster diagrams; it was written not only to analyze the equipartition conjecture of entropy production but also to explore the world of molecular classification.

## 4. Conclusions

From the present results and discussion the following conclusions can be drawn:

(1) The objective was to develop a structure–property relation for qualitative and quantitative prediction of chromatographic retention times of pesticides. Results of the present work contribute to relation prediction of pesticide residues, in food and environmental samples. Code TOPO allows fractal dimensions, and SCAP, solvation free energies and partition coefficient, which show that for a given atom energies and partitions are sensitive to the presence in the molecule of other atoms and functional groups. Fractal dimensions, partition coefficient, *etc.* differentiated pesticides. Parameters needed for co-ordination index are molar formation enthalpy, molecular weight and surface area. The morphological and co-ordination indices barely improved equations. Correlation between molecular area and weight points not only to a homogeneous molecular structure of pesticides, but also to the ability to predict and tailor their properties; the latter is nontrivial in environmental toxicology.
(2) Several criteria selected to reduce the analysis to a manageable quantity of pesticides, referred to structural and constituent characteristics related to nonplanarity, and the number of rings, and O, double-bonded S, N and Cl atoms. Classification was in agreement with the principal component analyses. Program MolClas is a simple, reliable, efficient and fast procedure for molecular classification based on equipartition conjecture of entropy production. It was written to analyze equipartition conjecture of entropy production and explore molecular-classification world.
(3) Periodic law does not satisfy physics-law status: (a) pesticides retentions are not repeated; perhaps chemical character; (b) order relations are repeated with exceptions. Analysis forces statement: Relations that any compound *p* has with its neighbour, *p* + 1, are approximately repeated for each period. Periodicity is not general; however, if substance natural order is accepted law must be phenomenological. Retention is not used in periodic-table generation and serves to validate it. The analysis of other properties would give an insight into the possible generality of the periodic table. The periodic classification was extended to phenylureas and sulphonylureas.

## References

[1] De Melo Abreu S., Caboni P., Cabras P., Garau V.L., Alves A. (2006). Validation and global uncertainty of a liquid chromatographic with diode array detection method for the screening of azoxystrobin, kresoxim-methyl, trifloxystrobin, famoxadone, pyraclostrobin and fenamidone in grapes and wine. Anal. Chim. Acta.

[2] Oliva J., Navarro S., Barba A., Navarro G. (1999). Determination of chlorpyrifos, penconazole, fenarimol, vinclozolin and metalaxyl in grapes, must and wine by on-line microextraction and gas chromatography. J. Chromatogr. A.

[3] Jiménez J.J., Bernal J.L., del Nozal M.J., Toribio L., Arias E. (2001). Analysis of pesticide residues in wine by solid-phase extraction and gas chromatography with electron capture and nitrogen–phosphorus detection. J. Chromatogr. A.

[4] Wang J.F., Luan L., Wang Z.Q., Jiang S.R., Pan C.P. (2007). Determination of 19 multi-residue pesticides in grape wine by gas chromatography-mass spectrometry with micro liquid-liquid extraction and solid phase extraction. Chin. J. Anal. Chem..

[5] Economou A., Botitsi H., Antoniou S., Tsipi D. (2009). Determination of multi-class pesticides in wines by solid-phase extraction and liquid chromatography-tandem mass spectrometry. J. Chromatogr. A.

[6] Hu Y., Liu W.M., Zhou Y.M., Guan Y.F. (2006). Determination of organophosphorous pesticide residues in red wine by solid phase microextraction-gas chromatography. Chin. J. Chromatogr..

[7] Wu J., Tragas C., Lord H., Pawliszyn J. (2002). Analysis of polar pesticides in water and wine samples by automated in-tube solid-phase microextraction coupled with high-performance liquid chromatography–mass spectrometry. J. Chromatogr. A.

[8] Bolaños P.P., Romero-González R., Frenich A.G., Vidal J.L.M. (2008). Application of hollow fibre liquid phase microextraction for the multiresidue determination of pesticides in alcoholic beverages by ultra-high pressure liquid chromatography coupled to tandem mass spectrometry. J. Chromatogr. A.

[9] Vinas P., Aguinaga N., Campillo N., Hernández-Córdoba M. (2008). Comparison of stir bar sorptive extraction and membrane-assisted solvent extraction for the ultra-performance liquid chromatographic determination of oxazole fungicide residues in wines and juices. J. Chromatogr. A.

[10] Anastassiades M., Lehotay S.J., Stajnbaher D., Schenck F.J. (2003). Fast and easy multiresidue method employing acetonitrile extraction/partitioning and “dispersive solid-phase extraction” for the determination of pesticide residues in produce. J. AOAC Int..

[11] Lehotay S.J., Zweigenbaum J. (2011). Mass Spectrometry in Food Safety.

[12] Cunha S.C., Lehotay S.J., Mastovska K., Fernandes J.O., Beatriz M., Oliveira P.P. (2007). Evaluation of the QuEChERS sample preparation approach for the analysis of pesticide residues in olives. J. Sep. Sci..

[13] Whelan M., Kinsella B., Furey A., Moloney M., Cantwell H., Lehotay S.J., Danaher M. (2010). Determination of anthelmintic drug residues in milk using ultra high performance liquid chromatography–tandem mass spectrometry with rapid polarity switching. J. Chromatogr. A.

[14] Wang X., Telepchak M.J. (2013). Determination of pesticides in red wine by QuEChERS extraction, rapid mini-cartridge cleanup and LC–MS–MS detection. LC·GC Eur..

[15] Blasco C., Picó Y. (2009). Prospects for combining chemical and biological methods for integrated environmental assessment. Trends Anal. Chem..

[16] de Umbuzeiro A.G. (2012). Guia de Potabilidade para Substàncias Químicas. http://www.abes-sp.org.br/arquivos/ctsp/guia_potabilidade.pdf.

[17] Moganti S., Richardson B.J., McClellan K., Martin M., Lam P.K.S., Zheng G.J. (2008). Use of the clam *Asaphis deflorata* as a potential indicator of organochlorine bioaccumulation in Hong Kong coastal sediments. Mar. Pollut. Bull..

[18] Barco-Bonilla N., Romero-González R., Plaza-Bolaños P., Garrido Frenich A., Martínez Vidal J.L. (2010). Analysis and study of the distribution of polar and non–polar pesticides in wastewater effluents from modern and conventional treatments. J. Chromatogr. A.

[19] Navarro A., Tauler R., Lacorte S., Barceló D. (2006). Chemometrical investigation of the presence and distribution of organochlorine and polyaromatic compounds in sediments of the Ebro River Basin. Anal. Bioanal. Chem..

[20] Navarro-Ortega A., Tauler R., Lacorte S., Barceló D. (2010). Occurrence and transport of PAHs, pesticides and alkylphenols in sediment samples along the Ebro River Basin. J. Hydrol..

[21] Toropov A.A., Toropova A.P., Benfenati E. (2009). QSPR modelling of the octanol/water partition coefficient of organometallic substances by optimal SMILES-based descriptors. Cent. Eur. J. Chem..

[22] Toropova A.P., Toropov A.A., Benfenati E., Gini G. (2011). QSAR models for toxicity of organic substances to *Daphnia magna* built up by using the CORAL freeware. Chem. Biol. Drug Des..

[23] Toropov A.A., Toropova A.P. (2014). Optimal descriptor as a translator of eclectic data into endpoint prediction: Mutagenicity of fullerene as a mathematical function of conditions. Chemosphere.

[24] Kar S., Roy K. (2013). Predictive chemometric modeling and three-dimensional toxicophore mapping of diverse organic chemicals causing bioluminescent repression of the bacterium genus *Pseudomonas*. Ind. Eng. Chem. Res..

[25] Roy K., Das R.N., Popelier P.L.A. (2014). Quantitative structure–activity relationship for toxicity of ionic liquids to *Daphnia magna*: Aromaticity *vs. * lipophilicity. Chemosphere.

[26] Torrens F. (2001). Free energy of solvation and partition coefficients in methanol–water binary mixtures. Chromatographia.

[27] Soria V., Campos A., Figueruelo J.E., Gómez C., Porcar I., García R., Potschka M., Dubin P.L. (1996). Modelling of stationary phase in size-exclusion chromatography with binary eluents. Strategies in Size Exclusion Chromatography.

[28] Torrens F., Soria V. (2002). Stationary-mobile phase distribution coefficient for polystyrene standards. Sep. Sci. Technol..

[29] Torrens F. (2003). A new chemical index inspired by biological plastic evolution. Indian J. Chem. Sect. A.

[30] Torrens F. (2004). A chemical index inspired by biological plastic evolution: Valence-isoelectronic series of aromatics. J. Chem. Inf. Comput. Sci..

[31] Torrens F., Castellano G. (2012). QSPR prediction of retention times of phenylurea herbicides by biological plastic evolution. Curr. Drug Saf..

[32] Torrens F., Castellano G., Roy K. Molecular categorization of phenylurea and sulphonylurea herbicides, pesticides and persistent organic pollutants. QSAR in Drug and Environmental Research.

[33] Torrens F., Castellano G. (2014). QSPR prediction of chromatographic retention times of pesticides: Partition and fractal indices. J. Environ. Sci. Health Part B.

[34] Varmuza K. (1980). Pattern Recognition in Chemistry.

[35] Benzecri J.P. (1984). L’Analyse des Données.

[36] Tondeur D., Kvaalen E. (1987). Equipartition of entropy production. An optimality criterion for transfer and separation processes. Ind. Eng. Chem. Fundam..

[37] Torrens F. (2003). Characterizing cavity-like spaces in active-site models of zeolites. Comput. Mater. Sci..

[38] (1989). IMSL. Integrated Mathematical Statistical Library (IMSL).

[39] Tryon R.C. (1939). A multivariate analysis of the risk of coronary heart disease in Framingham. J. Chronic Dis..

[40] Jarvis R.A., Patrick E.A. (1973). Clustering using a similarity measure based on shared nearest neighbors. IEEE Trans. Comput..

[41] Page R.D.M. (2000). Program TreeView.

[42] Huson D.H. (1998). SplitsTree: Analizing and visualizing evolutionary data. Bioinformatics.

[43] Hotelling H. (1933). Analysis of a complex of statistical variables into principal components. J. Educ. Psychol..

[44] Kramer R. (1998). Chemometric Techniques for Quantitative Analysis.

[45] Patra S.K., Mandal A.K., Pal M.K. (1999). State of aggregation of bilirubin in aqueous solution: Principal component analysis approach. J. Photochem. Photobiol. A.

[46] Jolliffe I.T. (2002). Principal Component Analysis.

[47] Xu J., Hagler A. (2002). Chemoinformatics and drug discovery. Molecules.

[48] Shaw P.J.A. (2003). Multivariate Statistics for the Environmental Sciences.

[49] Kaur M., Malik A.K., Singh B. (2013). Determination of phenylurea herbicides in tap water and soft drink samples by HPLC–UV and solid-phase extraction. LC·GC Eur..

[50] Can A., Yildiz I., Guvendik G. (2013). The determination of toxicities of sulphonylurea and phenylurea herbicides with quantitative structure–toxicity relationship (QSTR) studies. Environ. Toxicol. Pharmacol..

[51] Cabrera K., Altmaier S. (2013). High-resolution and ultra trace analysis of pesticides using silica monoliths. Int. Labmate.

[52] Forster S., Altmaier S. (2013). Qualitative LC–MS analysis of pesticides using monolithic silica capillaries and potential for assay of pesticides in kidney. LC·GC Eur..

[53] Nold M. (2009). Analytical standards for persistent organic pollutants. Analytix.

[54] Kaufmann A. (1975). Introduction à la Théorie des Sous-ensembles Flous.

[55] Cox E. (1994). The Fuzzy Systems Handbook.

[56] Kundu S. (1998). The min–max composition rule and its superiority over the usual max–min composition rule. Fuzzy Sets Sys..

[57] Lambert-Torres G., Pereira Pinto J.O., Borges da Silva L.E. (1999). Minmax techniques. Wiley Encyclopedia of Electrical and Electronics Engineering.

[58] Shannon C.E. (1948). A mathematical theory of communication: Part I, discrete noiseless systems. Bell Syst. Tech. J..

[59] Shannon C.E. (1948). A mathematical theory of communication: Part II, the discrete channel with noise. Bell Syst. Tech. J..

[60] White H. (1989). Neural network learning and statistics. AI Expert.

[61] Kullback S. (1959). Information Theory and Statistics.

[62] Iordache O., Corriou J.P., Garrido-Sánchez L., Fonteix C., Tondeur D. (1993). Neural network frames. application to biochemical kinetic diagnosis. Comput. Chem. Eng..

[63] Iordache O. (2011). Modeling Multi-Level Systems.

[64] Iordache O. (2012). Self-Evolvable Systems: Machine Learning in Social Media.

[65] Iordache O. (2014). Polytope Projects.

